# Supplementary material for: Edible Bird’s Nest (EBN) Ameliorates the Effects of Indomethacin (IMC)-Induced Embryo Implantation Dysfunction in Rats
Source: Biology (Basel). 2025 Feb 4;14(2):159. doi: 10.3390/biology14020159 (PMC11851620; doi:10.3390/biology14020159)
Supplement: Supplementary file 1 [file biology-14-00159-s001.zip › biology-3287002-supplementary.pdf]

biology

MDPI

Supplementary Materials:

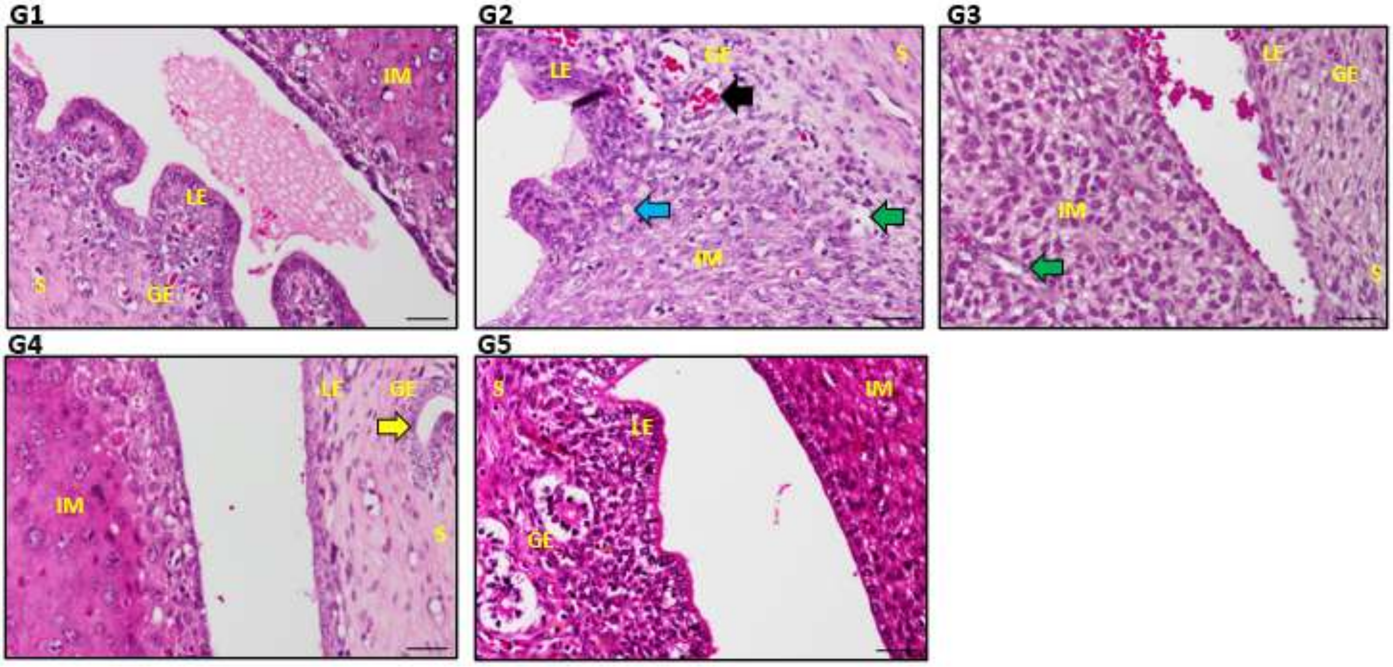

Figure S1. Impact of IMC exposure and EBN therapy on uterine histomorphology. (LE) signify luminal epithelium, (S) stroma and (GE) glandular epithelium. Normal uterine glands are indicated by yellow arrows, but congestion is indicated by black arrow, vacuolation is indicated by blue arrow and endometrial atrophy by green arrow in G2 and G3. Deterioration of LE cells by black arrow is manifest in G2 compared to treatment groups. H&E stains. Scale bar 50 µm.

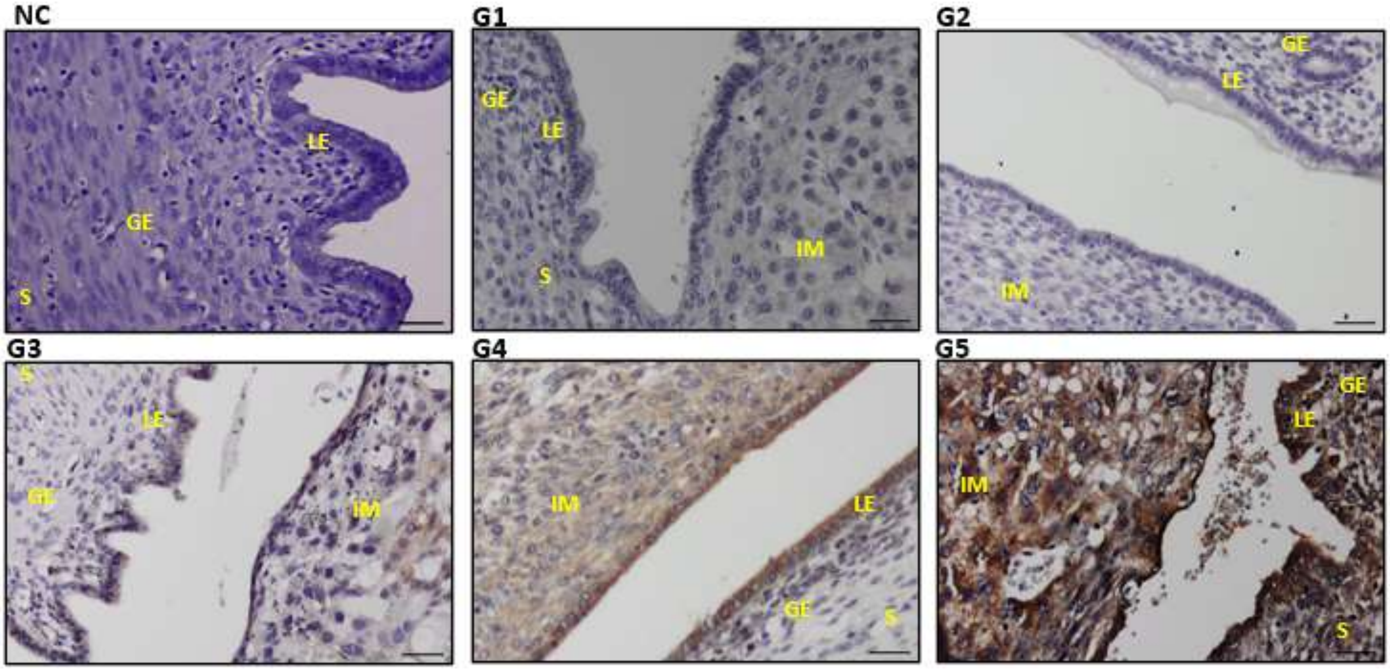

Figure S2. Photomicrograph sections of the uteri of rats of different experimental groups (G1, G2, G3, G4, and G5) treated with different doses of EBN showing expressions of epidermal growth factor (EGF). Stroma (S), the glandular epithelium (GE), and luminal epithelium (LE), with implantation sites (IM). Scale bar 50 µm.

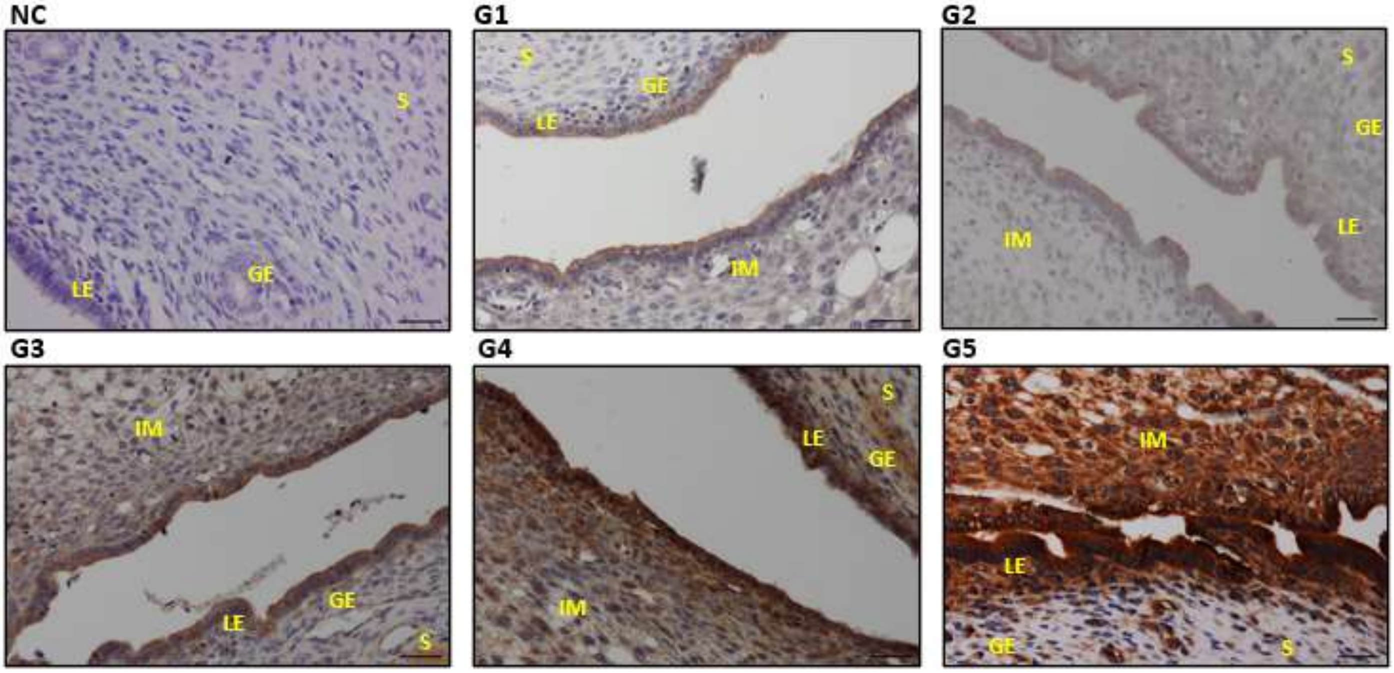

Figure S3. Photomicrograph sections of the uteri of rats of different experimental groups (G1, G2, G3, G4, and G5) treated with different doses of EBN showing expressions of EGFR. Stroma (S), the glandular epithelium (GE), and luminal epithelium (LE), with implantation sites (IM). Scale bar 50 µm.

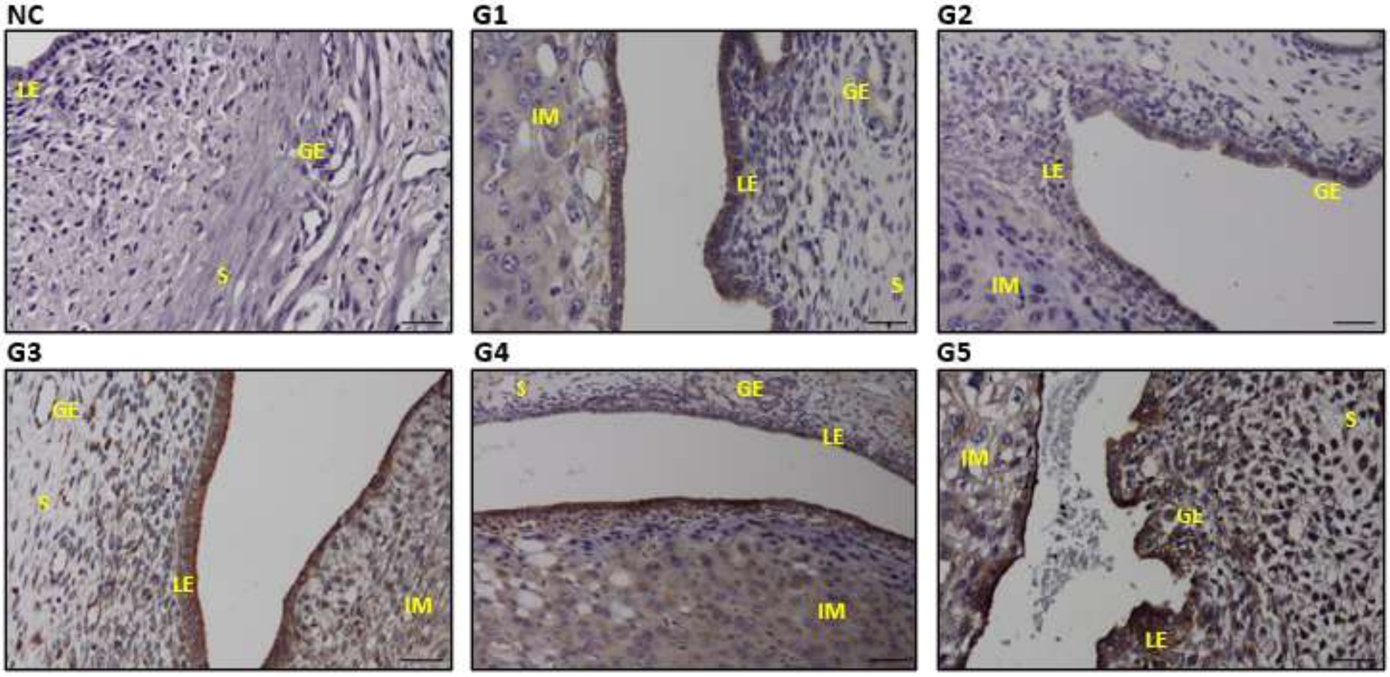

Figure S4. Photomicrograph sections of the uteri of rats of different experimental groups (G1, G2, G3, G4, and G5) treated with different doses of EBN showing expressions of vascular endothelial growth factor (VEGF). Stroma (S), the glandular epithelium (GE), and luminal epithelium (LE), with implantation sites (IM). Scale bar 50 µm.

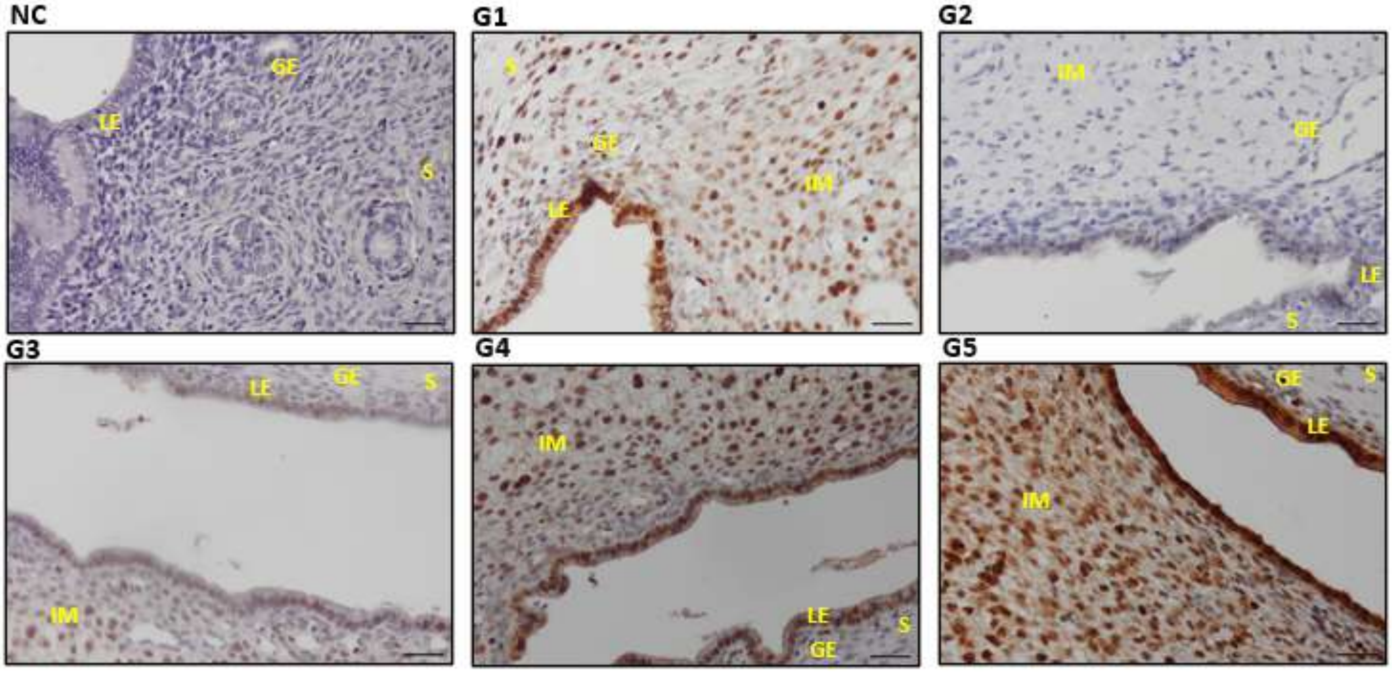

Figure S5. Photomicrograph sections of the uteri of rats of different experimental groups (G1, G2, G3, G4, and G5) treated with different doses of EBN showing expressions of proliferating cell nuclear antigen (PCNA). Stroma (S), the glandular epithelium (GE), and luminal epithelium (LE), with implantation sites (IM). Scale bar 50 µm.

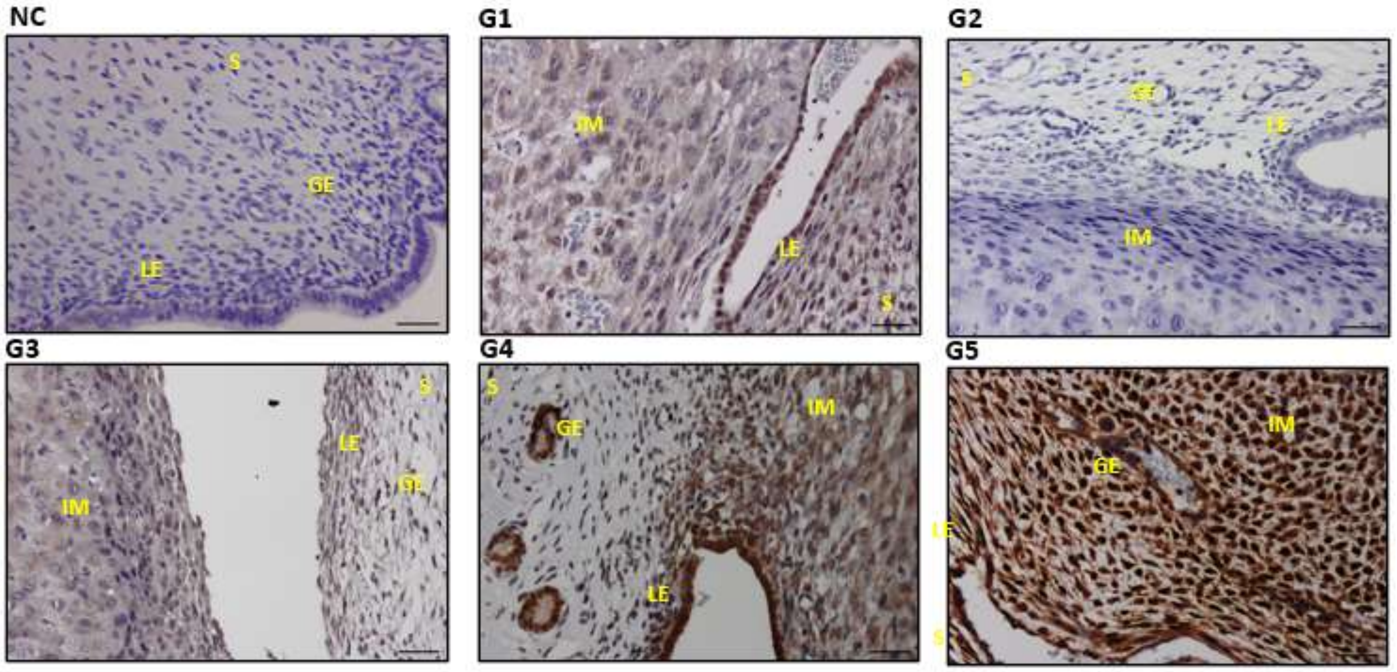

Figure S6. Photomicrograph sections of rat uteri of different experimental groups (G1, G2, G3, and G4). Estrogen receptor (E2R) was observed in all groups with the highest expression in G4 and G5. Stroma (S), the glandular epithelium (GE), and luminal epithelium (LE), with implantation sites (IM). Scale bar 50 μm.

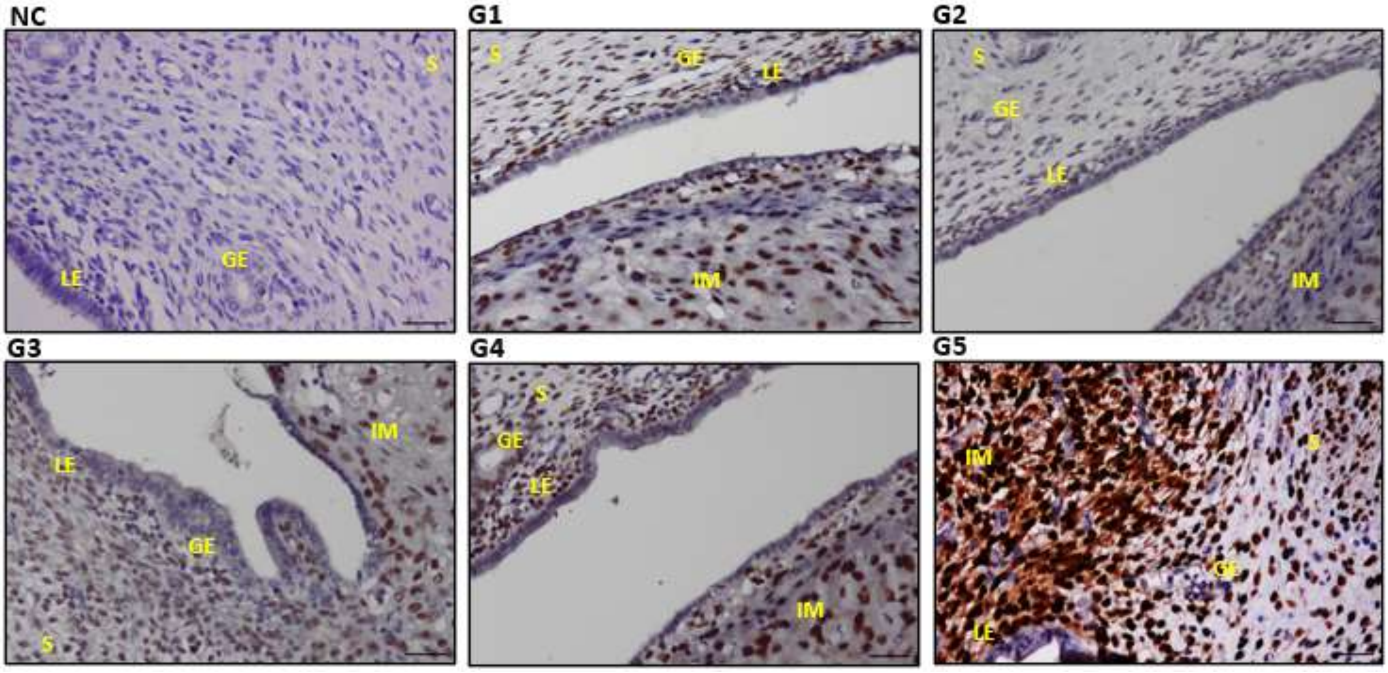

Figure S7. Photomicrograph sections of rat uteri of different experimental groups (G1, G2, G3, and G4) showing progesterone receptor (P4R) expressions. Note the higher expression of P4R in G5. Stroma (S), the glandular epithelium (GE), and luminal epithelium (LE), with implantation sites (IM). Scale bar 50 µm.
